# Supplementary material for: The Tumor Coagulome as a Transcriptional Target and a Potential Effector of Glucocorticoids in Human Cancers
Source: Cancers (Basel). 2023 Feb 28;15(5):1531. doi: 10.3390/cancers15051531 (PMC10001343; doi:10.3390/cancers15051531)
Supplement: Supplementary file 1 [file cancers-15-01531-s001.zip › cancers-2213006-supplementary.pdf]

Supplementary Data

The Tumor Coagulome as a Transcriptional Target and a Potential Effector of Glucocorticoids in Human Cancers

Floriane Racine, Christophe Louandre, Corinne Godin, Baptiste Chatelain, Stefan Prekovic, Wilbert Zwart, Antoine Galmiche and Zuzana Saidak

Supplementary Materials and Methods p2

Figure S1: Viability of OSCC cells treated with dexamethasone p3

Figure S2: Immunofluorescence labelling of PAI-1 in OSCC cells exposed to dexamethasone p4

Figure S3: Dose-response analysis of *F3*, *PLAU* and *SERPINE1* expression in OSCC cells exposed to dexamethasone p5

Figure S4: Effect of mifepristone, a GR antagonist, on the components of the coagulome p6

Figure S5: Clinical characteristics of OSCC tumors with high GR-activity/ high *SERPINE1* p7

Figure S6: TME analysis in PAAD tumors high in GR-activity and SERPINE1 p8

Table S1: OSCC groups according to GR-activity and *SERPINE1* expression p9

Table S2: GO terms most enriched in DEG in "High++" OSCC compared to other OSCC p14

Supplementary Materials and Methods

Cell culture and reagents

The human OSCC cell lines, PE/CA-PJ34 and PE/CA-PJ41, were purchased from ECACC (European Collection of Authenticated Cell Cultures). Cells were cultured in Dulbecco's Modified Eagle Medium (DMEM) supplemented with 10% fetal calf serum, 2 mM glutamine and streptomycin/penicillin. Lung carcinoma cell lines (A549, H2122, H1944, H1975 and H460 cells) were obtained from Rene Bernard's lab (Netherlands Cancer Institute, Netherlands) (Prekovic et al, 2021). Dexamethasone (D4902) and mifepristone (M8046) were purchased from Sigma Aldrich. Human recombinant TNFα (210-TA) was purchased from R&D Systems Biotechne. Cancer cell growth was evaluated with crystal violet staining (Sigma Aldrich).

Immunoblot analysis

Proteins were extracted and transferred to nitrocellulose membranes following immunoblot procedures and ECL reaction, as described elsewhere (Galmiche et al., 2010). Immunoblots were quantified using the Image J software (https://imagej.nih.gov/ij/download.html). The following primary antibodies were used: anti-TF (TF9-10H10, Sigma Aldrich), anti-uPA (Ab169754, Abcam), anti-PAI-1 (Ab66705, Abcam), anti-GR (12041S, Cell Signaling), and anti-β-actin (A5441, Sigma-Aldrich).

Fluorescence Microscopy

Cells grown on glass coverslips were fixed with 3.7% paraformaldehyde, permeabilized with 0.01% TritonX100, and stained with relevant antibodies. Coverslips were mounted in Mowiol (Calbiochem) and observed with a Nikon Eclipse TE2000U microscope equipped with a plan APO VC 60X / 1.40 objective under oil immersion.

Suppl. Tables and Figures

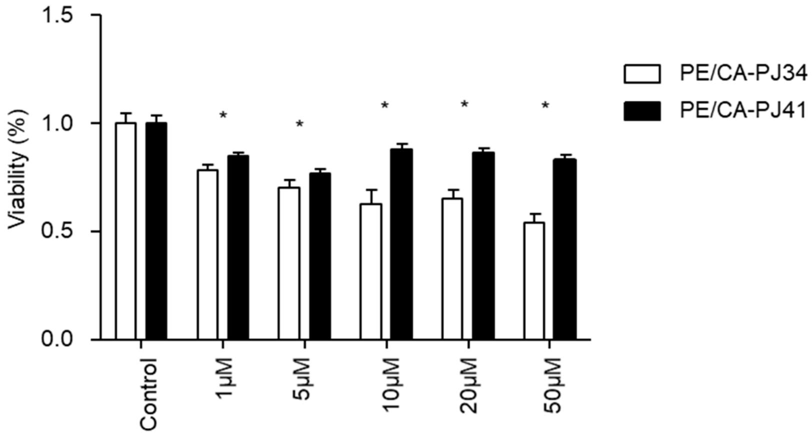

Figure S1. Viability of OSCC cells treated with dexamethasone

PE/CA-PJ34 and PE/CA-PJ41 were treated with dexamethasone at the indicated concentrations for 6 days. Cell viability was determined by Crystal Violet analysis. Results are represented as relative values, with control set as 1. Student's t test was used to compare each condition with control (*p<0.05).

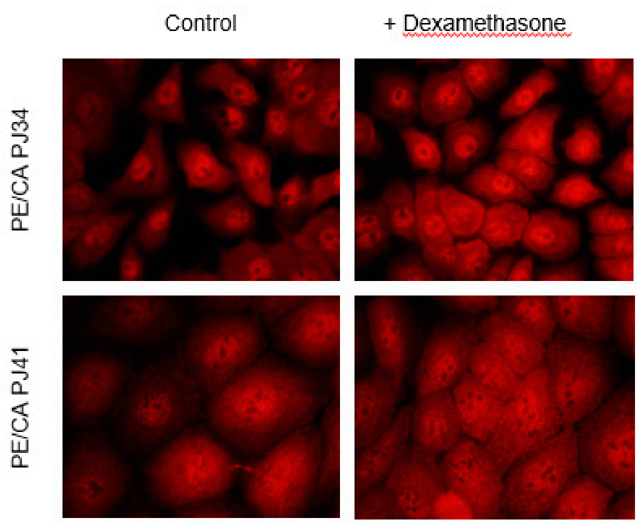

Figure S2. Immunofluorescence labelling of PAI-1 in OSCC cells exposed to dexamethasone.

PE/CA-PJ34 and PE/CA-PJ41 cells were exposed to dexamethasone (10 μM for 48 hours) and processed for PAI-1 staining.

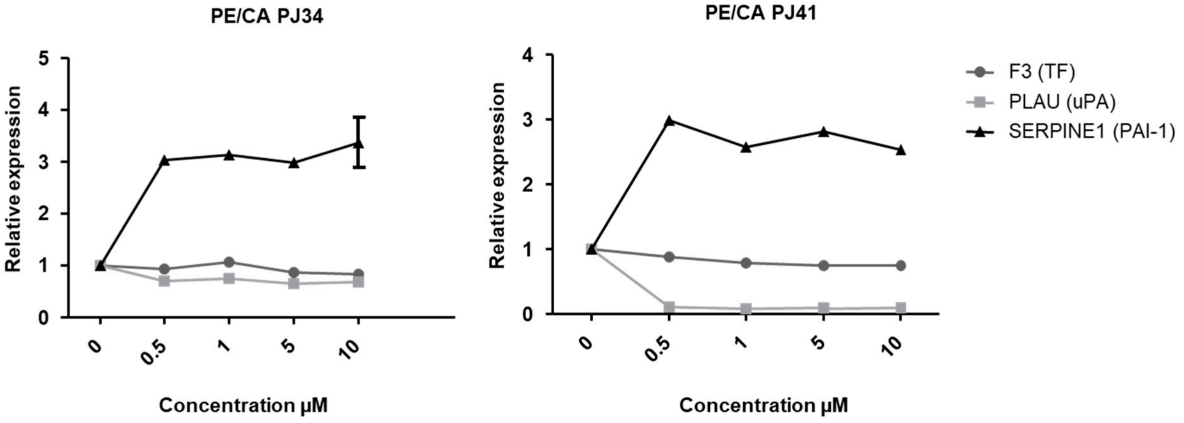

Figure S3. Dose-response analysis of *F3, PLAU and SERPINE1* expression in OSCC cells exposed to dexamethasone

QPCR analysis of the expression of *F3*, *PLAU* and *SERPINE1* in OSCC cells treated with dexamethasone (48 hour exposure) at increasing concentrations. Results are represented as relative expression, with control set as 1.

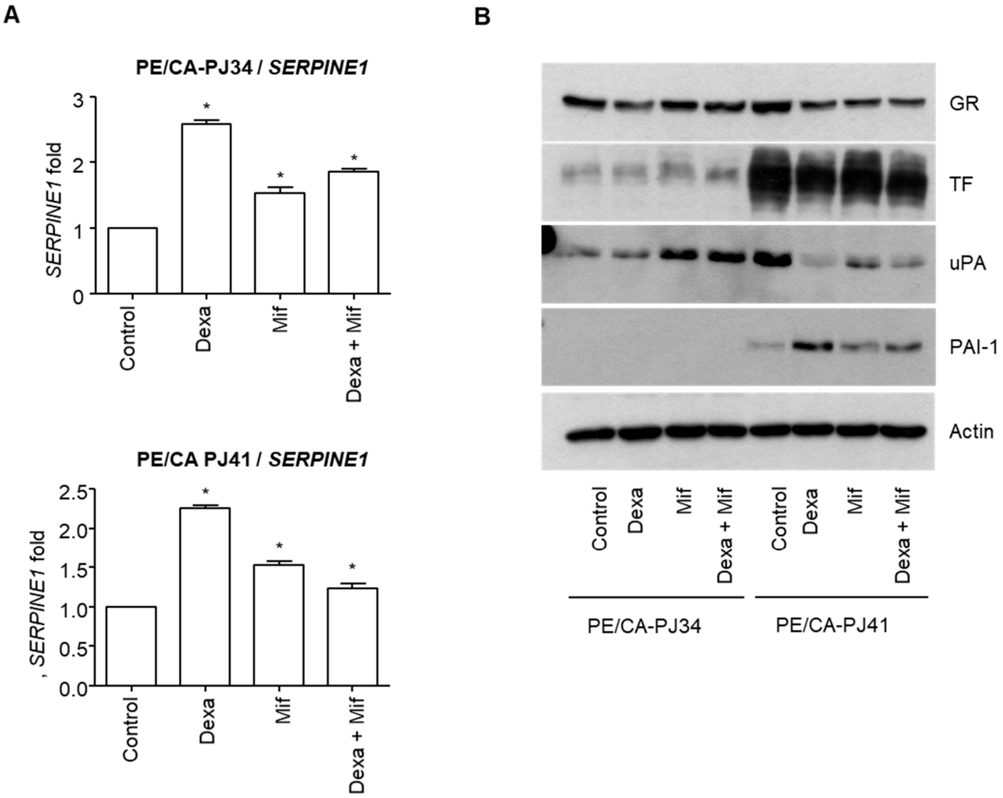

Figure S4. Effect of mifepristone, a GR antagonist, on the components of the coagulome

(A) QPCR analysis of the expression of *SERPINE1* in PE/CA-PJ34 and PE/CA-PJ41. Cells were treated with mifepristone (10 µM) for 24h followed by +/- dexamethasone (10 µM) for 48 hours. Student's T test was used to compare each condition with control (*p<0.05). (B) Immunoblot analysis of the expression of GR, TF, uPA and PAI-1 in OSCC cell lines treated with mifepristone (10 µM) +/- dexamethasone (10 µM). Actin was used as control.

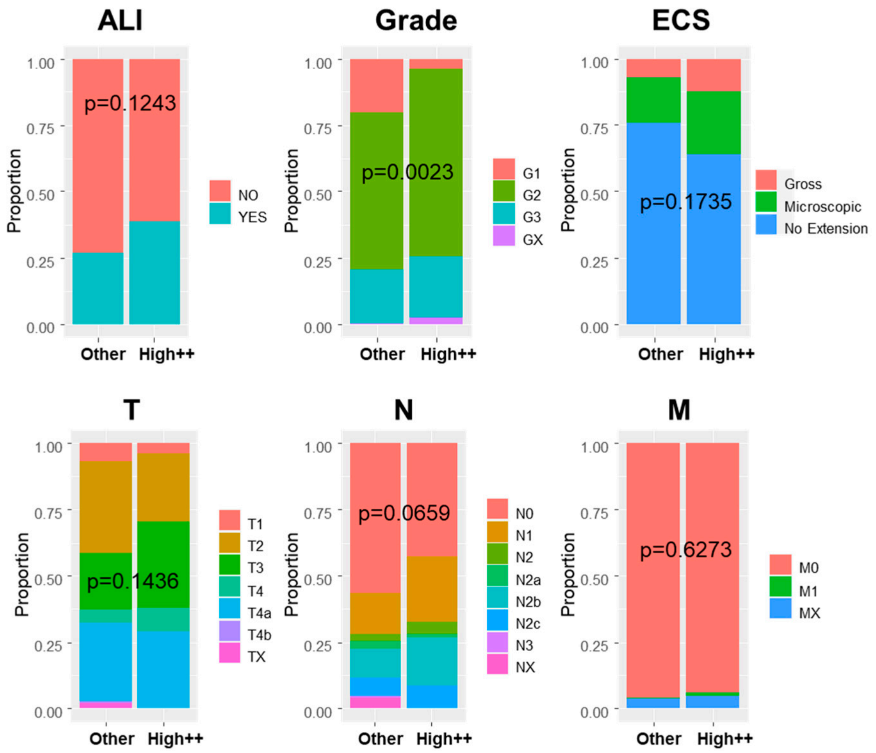

Figure S5. Clinical characteristics of OSCC tumors with high GR-activity/ high *SERPINE1*

(A) The subset of OSCC tumors with a “high GR activity” and “high *SERPINE1”* (i.e. “High++”, n=82) were compared with other OSCC regarding the occurrence of angiolymphatic invasion (ALI), tumor grade, extracapsular spread (ECS) and the TNM (tumor size (T), nodal involvement (N), metastasis (M)). Categories were compared using the Chi-squared test. $p<0.05$ was set as threshold for significance.

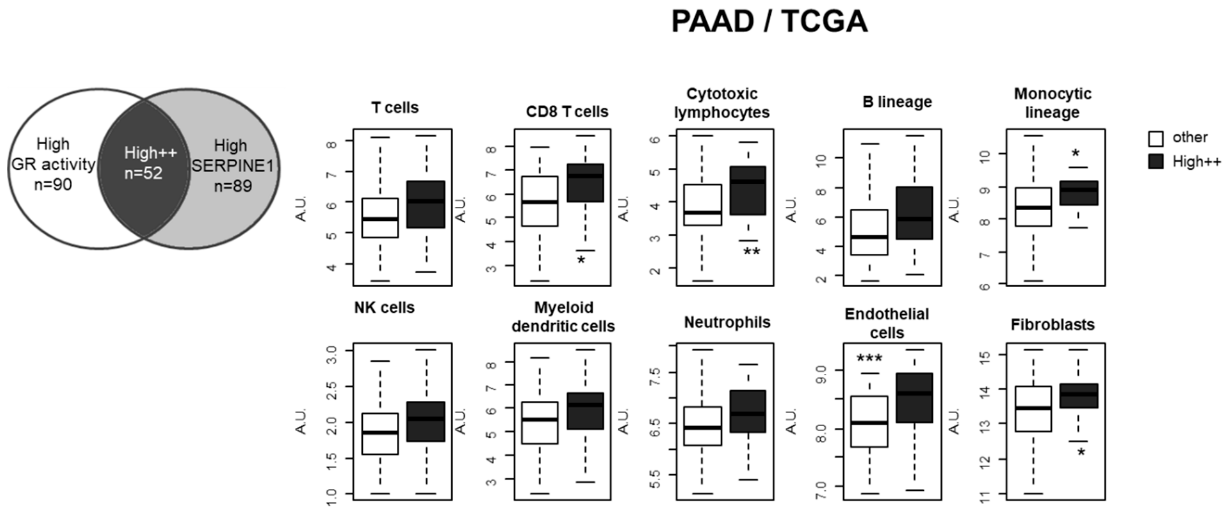

Figure S6. TME analysis in PAAD tumors high in GR-activity and *SERPINE1*

(A) The subset of PAAD tumors with a "high GR activity" (upper half) and high *SERPINE1* expression (upper half) (i.e. "High++") were identified n=52 (West et al., 2018). (B) The abundance of eight types of immune cells, and endothelial cells and fibroblasts in PAAD/TCGA tumors was stratified according to their GR-activity score and *SERPINE1* expression. Wilcoxon-Mann-Whitney was used to compare groups. *p<0.05 after FDR was set as threshold for significance.

Supplementary Table S1

OSCC groups according to GR-activity and *SERPINE1* expression

TCGA / OSCC sample	GR SCORE	GR-activity group	SERPINE1 group	High GR-activity / High SERPINE1
TCGA-4P-AA8J-01	-0,204	LOW	HIGH	other
TCGA-BA-4074-01	0,404	HIGH	LOW	other
TCGA-BA-4075-01	0,215	HIGH	HIGH	High++
TCGA-BA-5149-01	0,078	HIGH	HIGH	High++
TCGA-BA-5151-01	0,237	HIGH	LOW	other
TCGA-BA-5152-01	0,071	HIGH	LOW	other
TCGA-BA-5556-01	0,004	LOW	HIGH	other
TCGA-BA-5557-01	-0,063	LOW	HIGH	other
TCGA-BA-5558-01	0,016	LOW	LOW	other
TCGA-BA-6872-01	-0,173	LOW	HIGH	other
TCGA-BA-6873-01	0,152	HIGH	HIGH	High++
TCGA-BA-7269-01	-0,088	LOW	LOW	other
TCGA-BA-A6D8-01	-0,413	LOW	LOW	other
TCGA-BA-A6DB-01	0,115	HIGH	LOW	other
TCGA-BA-A6DD-01	0,012	LOW	HIGH	other
TCGA-BA-A6DE-01	0,034	HIGH	LOW	other
TCGA-BA-A6DG-01	0,088	HIGH	HIGH	High++
TCGA-BA-A6DJ-01	0,074	HIGH	HIGH	High++
TCGA-BB-4224-01	0,146	HIGH	LOW	other
TCGA-BB-7863-01	0,077	HIGH	LOW	other
TCGA-BB-7872-01	0,243	HIGH	HIGH	High++
TCGA-BB-8601-01	0,058	HIGH	LOW	other
TCGA-BB-A5HU-01	0,135	HIGH	HIGH	High++
TCGA-BB-A5HZ-01	0,046	HIGH	LOW	other
TCGA-BB-A6UO-01	0,034	HIGH	HIGH	High++
TCGA-C9-A47Z-01	0,053	HIGH	LOW	other
TCGA-C9-A480-01	0,053	HIGH	LOW	other
TCGA-CN-4725-01	0,008	LOW	HIGH	other
TCGA-CN-4726-01	0,087	HIGH	HIGH	High++
TCGA-CN-4728-01	0,008	LOW	HIGH	other
TCGA-CN-4729-01	-0,072	LOW	HIGH	other
TCGA-CN-4730-01	-0,051	LOW	LOW	other
TCGA-CN-4731-01	-0,034	LOW	LOW	other
TCGA-CN-4733-01	0,042	HIGH	HIGH	High++
TCGA-CN-4734-01	0,004	LOW	HIGH	other
TCGA-CN-4736-01	0,046	HIGH	LOW	other
TCGA-CN-4737-01	-0,102	LOW	LOW	other
TCGA-CN-4740-01	0,066	HIGH	HIGH	High++
TCGA-CN-4741-01	-0,013	LOW	LOW	other
TCGA-CN-4742-01	-0,025	LOW	HIGH	other
TCGA-CN-5358-01	0,079	HIGH	HIGH	High++
TCGA-CN-5359-01	0,084	HIGH	LOW	other
TCGA-CN-5364-01	-0,034	LOW	LOW	other
TCGA-CN-5367-01	0,279	HIGH	HIGH	High++
TCGA-CN-5369-01	0,158	HIGH	LOW	other
TCGA-CN-5370-01	0,283	HIGH	HIGH	High++
TCGA-CN-5373-01	-0,053	LOW	LOW	other
TCGA-CN-6011-01	-0,358	LOW	LOW	other
TCGA-CN-6013-01	-0,096	LOW	HIGH	other
TCGA-CN-6016-01	-0,356	LOW	HIGH	other
TCGA-CN-6017-01	0,017	LOW	LOW	other
TCGA-CN-6018-01	0,105	HIGH	HIGH	High++
TCGA-CN-6019-01	-0,069	LOW	HIGH	other
TCGA-CN-6020-01	0,035	HIGH	HIGH	High++
TCGA-CN-6024-01	0,031	HIGH	HIGH	High++
TCGA-CN-6994-01	-0,164	LOW	HIGH	other
TCGA-CN-6995-01	-0,057	LOW	HIGH	other
TCGA-CN-6996-01	0,127	HIGH	LOW	other
TCGA-CN-6998-01	0,041	HIGH	HIGH	High++
TCGA-CN-A49A-01	-0,032	LOW	LOW	other

TCGA-CN-A63V-01	-0,002	LOW	LOW	other
TCGA-CN-A642-01	0,155	HIGH	LOW	other
TCGA-CQ-5323-01	-0,032	LOW	LOW	other
TCGA-CQ-5324-01	-0,108	LOW	LOW	other
TCGA-CQ-5325-01	0,114	HIGH	LOW	other
TCGA-CQ-5326-01	0,037	HIGH	HIGH	High++
TCGA-CQ-5327-01	0,186	HIGH	LOW	other
TCGA-CQ-5329-01	0,225	HIGH	HIGH	High++
TCGA-CQ-5330-01	0,106	HIGH	HIGH	High++
TCGA-CQ-5331-01	0,189	HIGH	LOW	other
TCGA-CQ-5332-01	-0,006	LOW	HIGH	other
TCGA-CQ-5333-01	-0,222	LOW	LOW	other
TCGA-CQ-5334-01	-0,057	LOW	LOW	other
TCGA-CQ-6218-01	0,076	HIGH	LOW	other
TCGA-CQ-6219-01	0,089	HIGH	LOW	other
TCGA-CQ-6220-01	-0,126	LOW	LOW	other
TCGA-CQ-6221-01	0,016	LOW	LOW	other
TCGA-CQ-6222-01	0,055	HIGH	LOW	other
TCGA-CQ-6223-01	0,031	HIGH	LOW	other
TCGA-CQ-6224-01	0,048	HIGH	HIGH	High++
TCGA-CQ-6225-01	-0,064	LOW	LOW	other
TCGA-CQ-6227-01	0,069	HIGH	HIGH	High++
TCGA-CQ-6228-01	0,248	HIGH	LOW	other
TCGA-CQ-6229-01	0,115	HIGH	HIGH	High++
TCGA-CQ-7063-01	0,014	LOW	HIGH	other
TCGA-CQ-7065-01	-0,187	LOW	HIGH	other
TCGA-CQ-7067-01	0,016	LOW	HIGH	other
TCGA-CQ-7068-01	-0,042	LOW	LOW	other
TCGA-CQ-7069-01	-0,197	LOW	HIGH	other
TCGA-CQ-7071-01	0,223	HIGH	LOW	other
TCGA-CQ-7072-01	0,022	LOW	HIGH	other
TCGA-CQ-A4C6-01	-0,021	LOW	HIGH	other
TCGA-CQ-A4C7-01	0,117	HIGH	HIGH	High++
TCGA-CQ-A4C9-01	-0,128	LOW	LOW	other
TCGA-CQ-A4CA-01	-0,145	LOW	LOW	other
TCGA-CQ-A4CB-01	0,173	HIGH	HIGH	High++
TCGA-CQ-A4CD-01	0,001	LOW	HIGH	other
TCGA-CQ-A4CE-01	0,131	HIGH	LOW	other
TCGA-CQ-A4CG-01	0,138	HIGH	HIGH	High++
TCGA-CQ-A4CH-01	-0,008	LOW	HIGH	other
TCGA-CQ-A4CI-01	-0,001	LOW	LOW	other
TCGA-CR-6471-01	0,012	LOW	LOW	other
TCGA-CR-6484-01	0,043	HIGH	LOW	other
TCGA-CR-6488-01	-0,096	LOW	HIGH	other
TCGA-CR-6491-01	-0,159	LOW	HIGH	other
TCGA-CR-6492-01	0,058	HIGH	LOW	other
TCGA-CR-6493-01	-0,064	LOW	HIGH	other
TCGA-CR-7365-01	-0,013	LOW	HIGH	other
TCGA-CR-7367-01	0,125	HIGH	LOW	other
TCGA-CR-7368-01	0,069	HIGH	LOW	other
TCGA-CR-7369-01	0,208	HIGH	HIGH	High++
TCGA-CR-7372-01	-0,066	LOW	LOW	other
TCGA-CR-7373-01	0,177	HIGH	HIGH	High++
TCGA-CR-7376-01	0,079	HIGH	HIGH	High++
TCGA-CR-7377-01	0,072	HIGH	HIGH	High++
TCGA-CR-7379-01	0,116	HIGH	LOW	other
TCGA-CR-7380-01	0,021	LOW	LOW	other
TCGA-CR-7382-01	0,120	HIGH	HIGH	High++
TCGA-CR-7386-01	0,079	HIGH	HIGH	High++
TCGA-CR-7390-01	0,091	HIGH	HIGH	High++
TCGA-CR-7391-01	0,064	HIGH	LOW	other
TCGA-CR-7392-01	0,105	HIGH	HIGH	High++
TCGA-CR-7393-01	0,159	HIGH	LOW	other
TCGA-CR-7394-01	0,089	HIGH	LOW	other
TCGA-CR-7395-01	0,076	HIGH	LOW	other
TCGA-CR-7397-01	0,028	HIGH	HIGH	High++
TCGA-CR-7401-01	-0,036	LOW	LOW	other
TCGA-CV-5436-01	-0,093	LOW	LOW	other

TCGA-CV-5442-01	-0,061	LOW	LOW	other
TCGA-CV-5966-01	0,006	LOW	LOW	other
TCGA-CV-5970-01	0,097	HIGH	HIGH	High++
TCGA-CV-5971-01	0,235	HIGH	LOW	other
TCGA-CV-5973-01	-0,375	LOW	HIGH	other
TCGA-CV-5976-01	-0,092	LOW	HIGH	other
TCGA-CV-5977-01	-0,034	LOW	HIGH	other
TCGA-CV-5979-01	-0,139	LOW	HIGH	other
TCGA-CV-6003-01	-0,121	LOW	HIGH	other
TCGA-CV-6433-01	0,053	HIGH	LOW	other
TCGA-CV-6436-01	-0,299	LOW	LOW	other
TCGA-CV-6441-01	0,124	HIGH	LOW	other
TCGA-CV-6933-01	-0,212	LOW	HIGH	other
TCGA-CV-6934-01	0,145	HIGH	HIGH	High++
TCGA-CV-6936-01	-0,049	LOW	LOW	other
TCGA-CV-6937-01	-0,028	LOW	HIGH	other
TCGA-CV-6938-01	0,102	HIGH	LOW	other
TCGA-CV-6939-01	0,302	HIGH	LOW	other
TCGA-CV-6940-01	-0,085	LOW	HIGH	other
TCGA-CV-6941-01	0,029	HIGH	HIGH	High++
TCGA-CV-6942-01	0,064	HIGH	HIGH	High++
TCGA-CV-6945-01	-0,079	LOW	HIGH	other
TCGA-CV-6948-01	0,252	HIGH	LOW	other
TCGA-CV-6951-01	-0,005	LOW	LOW	other
TCGA-CV-6952-01	0,120	HIGH	HIGH	High++
TCGA-CV-6953-01	0,102	HIGH	LOW	other
TCGA-CV-6954-01	0,137	HIGH	LOW	other
TCGA-CV-6955-01	0,016	LOW	LOW	other
TCGA-CV-6956-01	-0,106	LOW	LOW	other
TCGA-CV-6959-01	0,122	HIGH	HIGH	High++
TCGA-CV-6960-01	-0,137	LOW	HIGH	other
TCGA-CV-6961-01	0,012	LOW	HIGH	other
TCGA-CV-7090-01	-0,119	LOW	HIGH	other
TCGA-CV-7091-01	0,104	HIGH	LOW	other
TCGA-CV-7095-01	-0,179	LOW	HIGH	other
TCGA-CV-7097-01	0,294	HIGH	HIGH	High++
TCGA-CV-7099-01	0,108	HIGH	HIGH	High++
TCGA-CV-7100-01	-0,019	LOW	LOW	other
TCGA-CV-7102-01	0,105	HIGH	HIGH	High++
TCGA-CV-7103-01	-0,115	LOW	LOW	other
TCGA-CV-7104-01	0,073	HIGH	HIGH	High++
TCGA-CV-7178-01	0,221	HIGH	HIGH	High++
TCGA-CV-7180-01	-0,122	LOW	LOW	other
TCGA-CV-7183-01	-0,131	LOW	LOW	other
TCGA-CV-7235-01	0,131	HIGH	HIGH	High++
TCGA-CV-7236-01	0,203	HIGH	HIGH	High++
TCGA-CV-7238-01	0,028	HIGH	HIGH	High++
TCGA-CV-7243-01	-0,122	LOW	HIGH	other
TCGA-CV-7252-01	0,099	HIGH	HIGH	High++
TCGA-CV-7253-01	0,014	LOW	LOW	other
TCGA-CV-7254-01	0,217	HIGH	LOW	other
TCGA-CV-7255-01	-0,093	LOW	HIGH	other
TCGA-CV-7263-01	-0,028	LOW	LOW	other
TCGA-CV-7407-01	0,145	HIGH	HIGH	High++
TCGA-CV-7409-01	-0,017	LOW	LOW	other
TCGA-CV-7411-01	0,000	LOW	HIGH	other
TCGA-CV-7413-01	-0,120	LOW	LOW	other
TCGA-CV-7414-01	0,010	LOW	HIGH	other
TCGA-CV-7416-01	0,001	LOW	HIGH	other
TCGA-CV-7423-01	-0,065	LOW	HIGH	other
TCGA-CV-7425-01	0,110	HIGH	LOW	other
TCGA-CV-7427-01	0,158	HIGH	LOW	other
TCGA-CV-7428-01	0,002	LOW	LOW	other
TCGA-CV-7429-01	0,171	HIGH	HIGH	High++
TCGA-CV-7432-01	0,060	HIGH	LOW	other
TCGA-CV-7434-01	-0,011	LOW	LOW	other
TCGA-CV-7435-01	0,167	HIGH	HIGH	High++
TCGA-CV-7438-01	0,022	LOW	HIGH	other

TCGA-CV-7446-01	0,589	HIGH	LOW	other
TCGA-CV-7568-01	0,258	HIGH	HIGH	High++
TCGA-CV-A45O-01	-0,158	LOW	LOW	other
TCGA-CV-A45P-01	0,084	HIGH	LOW	other
TCGA-CV-A45Q-01	-0,650	LOW	LOW	other
TCGA-CV-A45R-01	-0,033	LOW	LOW	other
TCGA-CV-A45T-01	0,024	LOW	HIGH	other
TCGA-CV-A45U-01	0,038	HIGH	LOW	other
TCGA-CV-A45V-01	0,050	HIGH	LOW	other
TCGA-CV-A45X-01	-0,002	LOW	LOW	other
TCGA-CV-A463-01	0,252	HIGH	LOW	other
TCGA-CV-A464-01	0,005	LOW	HIGH	other
TCGA-CV-A465-01	-0,058	LOW	LOW	other
TCGA-CV-A468-01	0,141	HIGH	LOW	other
TCGA-CV-A6JD-01	-0,187	LOW	HIGH	other
TCGA-CV-A6JE-01	-0,274	LOW	LOW	other
TCGA-CV-A6JN-01	-0,368	LOW	HIGH	other
TCGA-CV-A6JO-01	0,038	HIGH	HIGH	High++
TCGA-CV-A6JT-01	-0,452	LOW	HIGH	other
TCGA-CV-A6JU-01	-0,203	LOW	LOW	other
TCGA-CV-A6JY-01	0,010	LOW	LOW	other
TCGA-CV-A6JZ-01	0,180	HIGH	HIGH	High++
TCGA-CV-A6K0-01	0,164	HIGH	HIGH	High++
TCGA-CV-A6K2-01	0,060	HIGH	LOW	other
TCGA-CX-7082-01	0,043	HIGH	LOW	other
TCGA-CX-7085-01	0,097	HIGH	HIGH	High++
TCGA-CX-7086-01	0,123	HIGH	LOW	other
TCGA-CX-7219-01	0,271	HIGH	HIGH	High++
TCGA-CX-A4AQ-01	-0,305	LOW	LOW	other
TCGA-D6-6515-01	0,089	HIGH	LOW	other
TCGA-D6-6516-01	-0,035	LOW	LOW	other
TCGA-D6-6823-01	-0,072	LOW	LOW	other
TCGA-D6-6825-01	0,138	HIGH	HIGH	High++
TCGA-D6-6827-01	0,217	HIGH	LOW	other
TCGA-D6-8569-01	-0,049	LOW	HIGH	other
TCGA-D6-A4Z9-01	0,059	HIGH	LOW	other
TCGA-D6-A4ZB-01	-0,126	LOW	LOW	other
TCGA-D6-A6EM-01	-0,171	LOW	LOW	other
TCGA-D6-A6EN-01	-0,216	LOW	LOW	other
TCGA-D6-A6EO-01	0,006	LOW	LOW	other
TCGA-DQ-5624-01	0,096	HIGH	HIGH	High++
TCGA-DQ-5625-01	0,117	HIGH	HIGH	High++
TCGA-DQ-5630-01	0,086	HIGH	HIGH	High++
TCGA-DQ-5631-01	-0,042	LOW	HIGH	other
TCGA-DQ-7588-01	-0,147	LOW	LOW	other
TCGA-DQ-7592-01	0,116	HIGH	HIGH	High++
TCGA-F7-8489-01	0,007	LOW	HIGH	other
TCGA-F7-A50G-01	-0,027	LOW	LOW	other
TCGA-F7-A50J-01	0,025	LOW	HIGH	other
TCGA-F7-A61S-01	-0,016	LOW	HIGH	other
TCGA-F7-A61W-01	-0,081	LOW	HIGH	other
TCGA-F7-A624-01	0,279	HIGH	LOW	other
TCGA-H7-7774-01	0,083	HIGH	LOW	other
TCGA-H7-8501-01	0,355	HIGH	HIGH	High++
TCGA-H7-8502-01	0,143	HIGH	HIGH	High++
TCGA-H7-A6C4-01	0,172	HIGH	HIGH	High++
TCGA-HD-7831-01	-0,058	LOW	HIGH	other
TCGA-HD-7832-01	-0,067	LOW	LOW	other
TCGA-HD-7917-01	0,167	HIGH	LOW	other
TCGA-HD-8634-01	-0,001	LOW	LOW	other
TCGA-HD-8635-01	0,089	HIGH	HIGH	High++
TCGA-HD-A4C1-01	0,121	HIGH	LOW	other
TCGA-HD-A633-01	-0,017	LOW	HIGH	other
TCGA-HD-A6HZ-01	0,118	HIGH	HIGH	High++
TCGA-HD-A6I0-01	-0,476	LOW	LOW	other
TCGA-HL-7533-01	0,013	LOW	HIGH	other
TCGA-IQ-7631-01	-0,298	LOW	LOW	other
TCGA-IQ-7632-01	0,080	HIGH	LOW	other

TCGA-IQ-A61E-01	0,126	HIGH	HIGH	High++
TCGA-IQ-A61G-01	0,020	LOW	HIGH	other
TCGA-IQ-A61H-01	0,240	HIGH	LOW	other
TCGA-IQ-A61J-01	0,030	HIGH	LOW	other
TCGA-IQ-A6SG-01	0,032	HIGH	HIGH	High++
TCGA-IQ-A6SH-01	-0,161	LOW	LOW	other
TCGA-KU-A66T-01	0,101	HIGH	HIGH	High++
TCGA-KU-A6H8-01	0,207	HIGH	LOW	other
TCGA-MT-A51X-01	-0,029	LOW	HIGH	other
TCGA-MT-A67A-01	-0,113	LOW	LOW	other
TCGA-MT-A67D-01	0,098	HIGH	LOW	other
TCGA-MT-A67F-01	0,168	HIGH	HIGH	High++
TCGA-MT-A7BN-01	-0,220	LOW	HIGH	other
TCGA-P3-A5QA-01	-0,007	LOW	HIGH	other
TCGA-P3-A5QF-01	0,123	HIGH	LOW	other
TCGA-P3-A6T0-01	0,039	HIGH	HIGH	High++
TCGA-P3-A6T2-01	0,128	HIGH	HIGH	High++
TCGA-P3-A6T3-01	-0,007	LOW	HIGH	other
TCGA-P3-A6T4-01	0,165	HIGH	HIGH	High++
TCGA-P3-A6T5-01	-0,247	LOW	LOW	other
TCGA-P3-A6T6-01	-0,146	LOW	LOW	other
TCGA-P3-A6T7-01	0,155	HIGH	HIGH	High++
TCGA-P3-A6T8-01	0,031	HIGH	HIGH	High++
TCGA-QK-A64Z-01	0,111	HIGH	LOW	other
TCGA-QK-A652-01	-0,021	LOW	LOW	other
TCGA-QK-A6IG-01	-0,063	LOW	HIGH	other
TCGA-QK-A6IH-01	-0,368	LOW	LOW	other
TCGA-QK-A6II-01	-0,412	LOW	LOW	other
TCGA-QK-A6IJ-01	-0,550	LOW	LOW	other
TCGA-QK-A6VB-01	0,091	HIGH	LOW	other
TCGA-QK-A8Z7-01	0,050	HIGH	LOW	other
TCGA-QK-A8Z9-01	-0,027	LOW	HIGH	other
TCGA-QK-AA3K-01	0,059	HIGH	HIGH	High++
TCGA-RS-A6TO-01	-0,016	LOW	HIGH	other
TCGA-T2-A6WX-01	-0,106	LOW	HIGH	other
TCGA-T2-A6WZ-01	-0,363	LOW	LOW	other
TCGA-T2-A6X2-01	0,014	LOW	HIGH	other
TCGA-T3-A92N-01	0,041	HIGH	HIGH	High++
TCGA-UF-A719-01	-0,084	LOW	LOW	other
TCGA-UF-A71A-01	-0,027	LOW	HIGH	other
TCGA-UF-A71A-06	-0,022	LOW	LOW	other
TCGA-UF-A71B-01	0,042	HIGH	LOW	other
TCGA-UF-A71E-01	-0,118	LOW	LOW	other
TCGA-UF-A7JA-01	-0,137	LOW	LOW	other
TCGA-UF-A7JC-01	-0,106	LOW	HIGH	other
TCGA-UF-A7JD-01	0,109	HIGH	HIGH	High++
TCGA-UF-A7JO-01	0,122	HIGH	LOW	other
TCGA-UF-A7JS-01	-0,510	LOW	HIGH	other
TCGA-UF-A7JT-01	-0,004	LOW	HIGH	other
TCGA-UP-A6WW-01	-0,253	LOW	LOW	other
TCGA-WA-A7GZ-01	-0,048	LOW	LOW	other
TCGA-WA-A7H4-01	0,056	HIGH	LOW	other

Supplementary Table S2

GO terms most enriched in DEG in "High++" OSCC compared to other OSCC

GO term	Fold enrichment	p (FDR)
dendritic cell homeostasis	>100	0.03
negative regulation of endodermal cell differentiation	79	0.04
Interleukin 11 mediated signaling pathway	79	0.04
Positive regulation of monocyte aggregation	79	0.04
Positive regulation of platelet aggregation	30	0.02
Wound healing, spreading of epidermal cells	27	0.02
